# Lack of *GNAQ* and *GNA11* Germ-Line Mutations in Familial Melanoma Pedigrees with Uveal Melanoma or Blue Nevi

**DOI:** 10.3389/fonc.2013.00160

**Published:** 2013-06-28

**Authors:** Jason E. Hawkes, Jennifer Campbell, Daniel Garvin, Lisa Cannon-Albright, Pamela Cassidy, Sancy A. Leachman

**Affiliations:** ^1^Department of Dermatology and Huntsman Cancer Institute, University of Utah Health Sciences Center, Salt Lake City, UT, USA; ^*2*^Division of Genetic Epidemiology, Department of Internal Medicine, University of Utah School of Medicine, Salt Lake City, UT, USA; ^*3*^Department of Medicinal Chemistry L.S. Skagg’s Pharmacy, University of Utah Health Sciences Center, Salt Lake City, UT, USA; ^*4*^Department of Dermatology, Oregon Health & Science University, Portland, OR, USA

**Keywords:** *GNAQ*, *GNA11*, familial melanoma, germ-line, blue nevi, uveal melanoma

## Abstract

Approximately 10% of melanoma cases are familial, but only 25–40% of familial melanoma cases can be attributed to germ-line mutations in the *CDKN2A* – the most significant high-risk melanoma susceptibility locus identified to date. The pathogenic mutation(s) in most of the remaining familial melanoma pedigrees have not yet been identified. The most common mutations in nevi and sporadic melanoma are found in *BRAF* and *NRAS*, both of which result in constitutive activation of the MAPK pathway. However, these mutations are not found in uveal melanomas or the intradermal melanocytic proliferations known as blue nevi. Rather, multiple studies report a strong association between these lesions and somatic mutations in *Guanine nucleotide-binding protein G(q) subunit alpha* (*GNAQ*), *Guanine nucleotide-binding protein G(q) subunit alpha-11* (*GNA11*), and *BRCA1-associated protein-1* (*BAP1*). Recently, germ-line mutations in BAP1, the gene encoding a tumor suppressing deubiquitinating enzyme, have been associated with predisposition to a variety of cancers including uveal melanoma, but no studies have examined the association of germ-line mutations in *GNAQ* and *GNA11* with uveal melanoma and blue nevi. We have now done so by sequencing exon 5 of both of these genes in 13 unique familial melanoma pedigrees, members of which have had either uveal or cutaneous melanoma and/or blue nevi. Germ-line DNA from a total of 22 individuals was used for sequencing; however no deleterious mutations were detected. Nevertheless, such candidate gene studies and the discovery of novel germ-line mutations associated with an increased MM susceptibility can lead to a better understanding of the pathways involved in melanocyte transformation, formulation of risk assessment, and the development of specific drug therapies.

## Introduction

Approximately 10% of melanoma cases are familial (Goldstein and Tucker, 2001). However, only 25–40% of familial melanoma cases can be specifically attributed to pathogenic germ-line mutations in *cyclin-dependant kinase inhibitor 2A* (*CDKN2A/p16*) – the most significant high-risk melanoma susceptibility gene identified to date (Goldstein and Tucker, 2001; Eliason et al., 2006; Leachman et al., 2009). Two other genes, *cyclin-dependant kinase 4* (*CDK4*) and *alternate reading frame* (*ARF*) have been confirmed as additional high penetrance melanoma predisposition genes, but account for less than 5% of hereditary melanoma families worldwide (Leachman et al., 2009). GWAS analyses have identified several additional moderate and low-penetrance melanoma predisposition genes but these contribute a small percentage to the overall genetic risk (Amos et al., 2011). Therefore, the majority of melanoma cases do not carry a known genetic mutation that accounts for their increased risk of melanoma (Hayward, 2003).

The most common mutations in sporadic melanoma are those of *v-Raf murine sarcoma viral oncogene homolog B1* (*BRAF*) and *neuroblastoma RAS viral oncogene homolog* (*NRAS*), both of which result in constitutive activation of the MAPK pathway and subsequent activation of pro-proliferative genes such as *cyclin-D1* (*CCND1*) (Onken et al., 2008). However, these mutations do not characterize all melanocytic neoplasms or intradermal melanocytic proliferations such as uveal melanoma and blue nevi, respectively (Saldanha et al., 2004). Rather, multiple studies have reported a strong association between these melanocytic lesions and somatic *guanine nucleotide-binding protein G(q) subunit alpha* (*GNAQ*), *guanine nucleotide-binding protein G(q) subunit alpha-11* (*GNA11*), and *BRCA1-associated protein-1* (*BAP1*) mutations in the absence of *BRAF*, *NRAS*, and *KIT* mutations (Harbour et al., 2010; Van Raamsdonk et al., 2010). Recently, germ-line mutations in *BAP1*, the gene encoding a tumor suppressing deubiquitinating enzyme, have been associated with predisposition to a variety of cancers including uveal and cutaneous melanoma as well as mesothelioma (Abdel-Rahman et al., 2011; Testa et al., 2011; Harbour, 2012; Wadt et al., 2012), but no studies have examined the association of germ-line mutations in *GNAQ* and *GNA11* with uveal melanoma and blue nevi.

**Figure 1:**
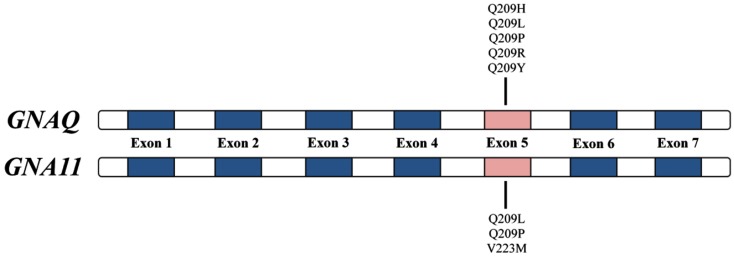
**Most common activating exon 5 *GNAQ* and *GNA11* gene mutations**.

*GNAQ* (OMIM ID 600998), found on chromosome 9q21, and *GNA11* (OMIM ID 139313), found on chromosome 19p13.3, encode the G-protein α subunit of heterotrimeric GTP-binding proteins and couple to the endothelin B receptor in melanocytes – a required signaling pathway for melanocyte development (Dong et al., 1995; Shin et al., 1999). The *GNAQ* and *GNA11* mutations associated with uveal melanoma and blue nevi occur almost exclusively in exon 5 (most commonly Q209L; Figure 1) and involve the glutamine residue within the *ras*-like domain, which plays an essential role in the GTP hydrolysis activity of this gene’s protein products (Van Raamsdonk et al., 2008, 2010). Activating *GNAQ* and *GNA11* mutations, such as those at codon 209, lock the GTP-binding protein in their active, GTP-bound state resulting in constitutive activation of the MAPK pathway in the absence of *BRAF* and *NRAS* mutations (Van Raamsdonk et al., 2008). In mice, these activating mutations ultimately function as oncogenes resulting in proliferation of intradermal and transformed melanocytes (Van Raamsdonk et al., 2004, 2008). These mouse studies provide a genetic basis to help explain why intradermal melanocytic proliferations affecting the conjunctiva and periorbital skin (nevi of Ota) are a risk factor for uveal melanoma (Van Raamsdonk et al., 2008). The work of Van Raamsdonk et al. and others suggest that mutations in *GNAQ* and *GNA11* represent an early event in the development of melanocytic tumors and may contribute directly to the increased melanoma risk in hereditary melanoma families that also have an increased incidence of uveal melanoma and/or blue nevi.

We hypothesized that an increased melanoma risk in familial melanoma families with uveal melanoma and/or blue nevi is due to *GNAQ* and *GNA11* germ-line mutations in exon 5 which result in constitutive activation of the MAPK pathway. To test this hypothesis, we investigated the frequency of *GNAQ* and *GNA11* exon 5 germ-line mutations in 22 patients who had a personal history of uveal melanoma and/or blue nevi from a total of 13 unique familial melanoma pedigrees previously identified as being high-risk for the development of melanoma.

## Materials and Methods

### Study subjects

Through the Familial Melanoma Research Clinic at the Huntsman Cancer Institute, we identified 22 study subjects who had a personal history of uveal melanoma and/or blue nevi and were also members of a pedigree with familial melanoma (defined as ≥2 first-degree relatives with a history of melanoma or pancreatic cancer or ≥3 family member with a history of melanoma of any relationship) (Supplementary Material). This study was approved by the University of Utah’s Institutional Review Board (IRB# 7616), which also acts as the University’s Ethical Review Board.

### Nucleic acid isolation and PCR amplification

From each of the 22 study subjects, archived DNA for genetic analysis was obtained using peripheral whole blood collected in Acid Citrate Dextrose (ACD) Venous Blood Vacuum Collection Tubes. Genomic DNA was isolated using Gentra Puregene Kit (Qiagen Inc.). DNA purity and concentration was determined using the NanoDrop 2000 Spectrophotometer (Thermo Scientific). PCR amplification of exon 5 of *GNAQ* and *GNA11* was performed using HotStarTaq DNA Polymerase (Qiagen Inc.) with the primers listed in Table 1. All PCR primers were designed and purchased from the University of Utah’s DNA Sequencing and Genomics Core Facility. For all PCR reactions, 1 μL of genomic DNA [50 ng/μL] was used as a PCR template in 20 μL total reaction volume containing 2 μL 10× PCR Buffer (Denville Scientific, Inc.), 1.6 μL 2.5 mM dNTP Mix (Invitrogen), 1 μL of each forward and reverse primer [10 mM], 13.2 μL H2O, and 0.2 μL Hot-Start Taq (5 U/mL) (Denville Scientific, Inc.). The conditions for PCR amplification were 95°C for 5 min followed by 35 cycles of 95°C for 15 s, 60°C for 20 s, and 72°C for 20 s. Following amplification, 3 μL of product and 1 μL of 1 kb Plus DNA Ladder (Invitrogen) were loaded and run on 1% agarose gels at 100 V for 30 min and DNA bands were visualized on a UV transilluminator after ethidium bromide staining. All PCR products where then purified using the ExoSAP-IT PCR Cleanup Protocol (Affymetrix/USB). PCR products where then purified using the ExoSAP-IT PCR Cleanup Protocol (Affymetrix/USB).

**Table 1:** **PCR primers used for the mutation profiling of *GNAQ*, *GNA11*, and *BAP1***.

Gene	Exon	Forward primer sequence 5′–3′	Reverse primer sequence 5′–3′
*GNAQ*	5	TTTCCCTAAGTTTGTAAGTAGTGCT	AAGTTCACTCCATTCCCCAC
*GNA11*	5	AGCCGATGTCAGTCTGGTGT	AAGGCAGAGGGAATCAGAGG
*BAP1*	4	AGTGATGACGCAGTGCAAAG	CTCCATTTCCACTTCCCAAG
	5	TGTCCAGATATGACTGACCTG	ATGTGGTAGCATTCCCAGTG
	6–7	TCTGAAGCTTTGCCTTCCAC	GCCACTGGGTACCACATACC
	8	TGTCTTCCTTCCCACTCCTG	TGGATACTCTCTGTCCCTCCC
	9	CTCAACCTGATGGCGGG	AATGCAGGGAGGGTTGG
	10	TTCCTTTAGGTCCTCAGCCC	AAAAGACTTTCCCTGTTTAGG
	11	TCTCTGGGAAGTGCTGGTTC	CATGGGAAAATTGCCTGTTG
	12	CCGAGCAGCACTTGTTTG	GATCCGAAGCACCTAGAACC
	13	AGCCATTCTGGGTACTGCTG	GAGTGCAGGACACTTTGTGG
	15–16	CTGCCTATTGCTCGTGGG	CAAGGTCTGCTCAAGCCTC
	17	ACAGGGAGGGCCATGAG	TACTGGGAAAAGGGGAAGTG

### Genetic analysis of study subjects

The University of Utah’s DNA Sequencing and Genomics Core Facility performed sequencing reactions in both directions using Big Dye Terminator chemistry on an ABI Prism 3700 DNA analyzer. Sequences were aligned and analyzed for single nucleotide polymorphisms and/or mutations with respect to published reference sequences found in the UCSC Genome Browser using Sequencher 4.5 software (Ann Arbor, MI, USA). Analysis of *CDKN2A* was performed by sequencing the 3 exons plus 95 non-coding base pairs of *p16* (Myriad Genetics, Salt Lake City, UT, USA), as well as exon 1-beta which codes for a portion of *p14 ARF* (Gene Dx, Gaithersburg, MD, USA). *CDK4* analysis was carried out by sequencing exon 2 and flanking splice sites (Gene Dx, Gaithersburg, MD, USA).

## Results

Of these 22 study subjects (Table 2), 14 had a personal history of cutaneous melanoma, 3 had a personal history of uveal melanoma (although there are four pedigrees that have individuals with uveal melanoma), 13 had a personal history of blue nevi, and 5 had a personal history of cutaneous melanoma as well as blue nevi. The 22 study subjects were from a total of 13 unique familial melanoma pedigrees (Supplementary Material). Of the 22 samples studied, all were wild-type at exon 5 for both *GNAQ* and *GNA11*. The results collected from this subset of high-risk melanoma families indicate that the inherited risk observed in these hereditary melanoma families is not due to activating germ-line mutations in exon 5 of *GNAQ* and *GNA11*.

**Table 2:** **Study subject characteristics and summary of sequencing results for *CDKN2A*, *CDK4*, and exon 5 of *GNAQ* and *GNA11***.

Study Subject ID	Pedigree ID	Individual	Individual	Individual	Individual	Individual	Pedigree	Pedigree	Pedigree	Pedigree	Pedigree
		
		History of cutaneous melanoma	History of uveal melanoma	History of blue nevi	*GNAQ* exon 5 genotyping results	*GNA11* exon 5 genotyping results	Number of cutaneous melanomas	Number of blue nevi	Number of uveal melanoma	*CDKN2A* genotyping results	*CDK4* genotyping results
1	A	Yes	No	Yes	WT	WT	7	1	0	WT	WT
2	B	Yes	No	Yes	WT	WT	17	1	0	WT	WT
3	C	Yes	Yes	No	WT	WT	3	0	1	WT	WT
4	D	Yes	No	Yes	WT	WT	9	1	0	WT	WT
5	E	Yes	No	Yes	WT	WT	42	1	0	WT	WT
6	F	No	No	Yes	WT	WT	4	1	0	WT	WT
7	G	Yes	Yes	No	WT	WT	8	0	1	WT	WT
8	H	No	No	Yes	WT	WT	4	3	1	A148T G > A (p16)	WT
9	H	No	No	Yes	WT	WT	–	–	–	A148T G > A (p16)	WT
10	H	Yes	No	No	WT	WT	–	–	–	A148T G > A (p16)	WT
11	H	No	No	Yes	WT	WT	–	–	–	A148T G > A (p16)	WT
12	H	Yes	No	No	WT	WT	–	–	–	A148T G > A (p16)	WT
13	I	No	No	Yes	WT	WT	3	2	0	Not determined	Not determined
14	J	Yes	No	Yes	WT	WT	6	2	0	Not determined	Not determined
15	J	No	No	Yes	WT	WT	–	–	–	Not determined	Not determined
16	K	No	No	Yes	WT	WT	3	1	0	WT	WT
17	L	No	No	Yes	WT	WT	2	1	0	Not determined	Not determined
18	M	Yes	No	No	WT	WT	5	0	1	WT	WT
19	M	Yes	No	No	WT	WT	–	–	–	WT	WT
20	M	Yes	Yes	No	WT	WT	–	–	–	WT	WT
21	M	Yes	No	No	WT	WT	–	–	–	WT	WT
22	M	Yes	No	No	WT	WT	–	–	–	WT	WT

*WT, wild-type*.

The studied pedigrees were previously determined to lack germ-line mutations in *CDKN2A*, *p16*, *ARF*, and *CDK4*, with the exception of three pedigrees for which sequencing data could not be obtained. These sequencing results are listed in Table 2. Additionally, screening for *BAP1* mutations in exons 9 and 12 was performed on all 22 study subjects. Sequencing of exons 4–13 and 15–17 of *BAP1* was performed on Study Subjects 3 (pedigree C) and 7 (pedigree G), both of whom had a personal history of uveal and cutaneous melanoma. In all instances, no *BAP1* mutations were found.

## Discussion

Malignant melanoma is a devastating malignancy for which few effective targeted treatments (e.g., *BRAF* inhibitors) are available. The major aim of the current investigation was to determine whether or not germ-line mutations in exon 5 of *GNAQ* and *GNA11* represent an early event in the development of melanocytic tumors and/or potential genetic biomarkers associated with the increased melanoma risk observed in hereditary melanoma families that lack other known pathogenic germ-line mutations. The lack of *GNAQ* and *GNA11* germ-line mutations in familial melanoma pedigrees with an increased incidence of uveal melanoma and blue nevi further is supportive of the importance of sporadic mutations in these genes in blue nevi and uveal melanoma as previously published. Nevertheless, the functional consequence of activating *GNAQ* and *GNA11* mutations on the MAPK pathway highlights an important concept: that specific gene mutations may result in an alternate route of MAPK pathway activation and subsequent melanocyte proliferation in the absence of more common gene mutations such as those in *BRAF*, *NRAS*, and *KIT*.

The major limitation of this study is the small sample size and limited number of familial melanoma pedigrees (*n* = 13) and uveal melanoma cases (*n* = 4). Therefore, it is necessary that further studies be done and our hypothesis be considered in a larger sample size before any final conclusion can be drawn. However, to our knowledge, this is one of the largest studies to date looking specifically at germ-line mutations in familial melanoma pedigrees with uveal melanoma and/or blue nevi. This study is also a retrospective study and is not designed to elucidate the complex interaction between specific gene mutations, phenotype characteristics, and MM susceptibility. Additionally, this study is not a complete survey of all of the genes thought to confer an increased familial melanoma risk and the screening for germ-line mutations in *CDKN2A*, *p16*, *ARF*, *CDK4*, and *BAP1* was incomplete. Subsequent studies are therefore necessary to determine the genetic basis for the increased risk of MM seen in the families included in this study. Finally, our study was limited to exon 5 of *GNAQ* and *GNA11*. It is, however, possible that an activating mutation outside of the *ras*-like domain may be present in the families we studied though this is unlikely given that activating mutations are found almost exclusively in exon 5 as mentioned above (Van Raamsdonk et al., 2008, 2010).

In summary, we report the absence of germ-line mutations in exon 5 of *GNAQ* and *GNA11* in familial melanoma pedigrees with an increased incidence of uveal melanoma and/or blue nevi. Melanoma’s high incidence and poor treatment outcomes as well as the high number of familial melanoma cases lacking known pathogenic germ-line mutations, underscores the importance of future studies using a candidate gene approach when phenotypic annotation is available. Such candidate gene studies and the discovery of novel germ-line mutations associated with an increased MM susceptibility can lead to a better understanding of the pathways involved in melanocyte transformation, formulation of risk assessment, and the development of specific drug therapies. Additionally, our study not only shows that our families don’t have known genetic mutations accounting for their increased melanoma risk, but also suggests that the genetic cause of familial ocular melanoma and blue nevi is yet to be discovered and that further investigation of these families could lead to identification of new targets for ocular melanoma. Further, a better understanding of the genetic basis observed in the inherited risk associated with familial melanoma may yield insights into the molecular pathogenesis of sporadic melanoma and, ultimately, improved methods of detection and treatment.

## Conflict of Interest Statement

The authors declare that the research was conducted in the absence of any commercial or financial relationships that could be construed as a potential conflict of interest.

## Supplementary Material

The Supplementary Material for this article can be found online at: http://www.frontiersin.org/Cancer_Genetics/10.3389/fonc.2013.00160/abstract
