# Supplementary material for: Lack of GNAQ and GNA11 Germ-Line Mutations in Familial Melanoma Pedigrees with Uveal Melanoma or Blue Nevi
Source: Front Oncol. 2013 Jun 28;3:160. doi: 10.3389/fonc.2013.00160 (PMC3695489; doi:10.3389/fonc.2013.00160)
Supplement: Supplementary file 1 [file 48332__Presentation_1.PPTX]

## Slide 1
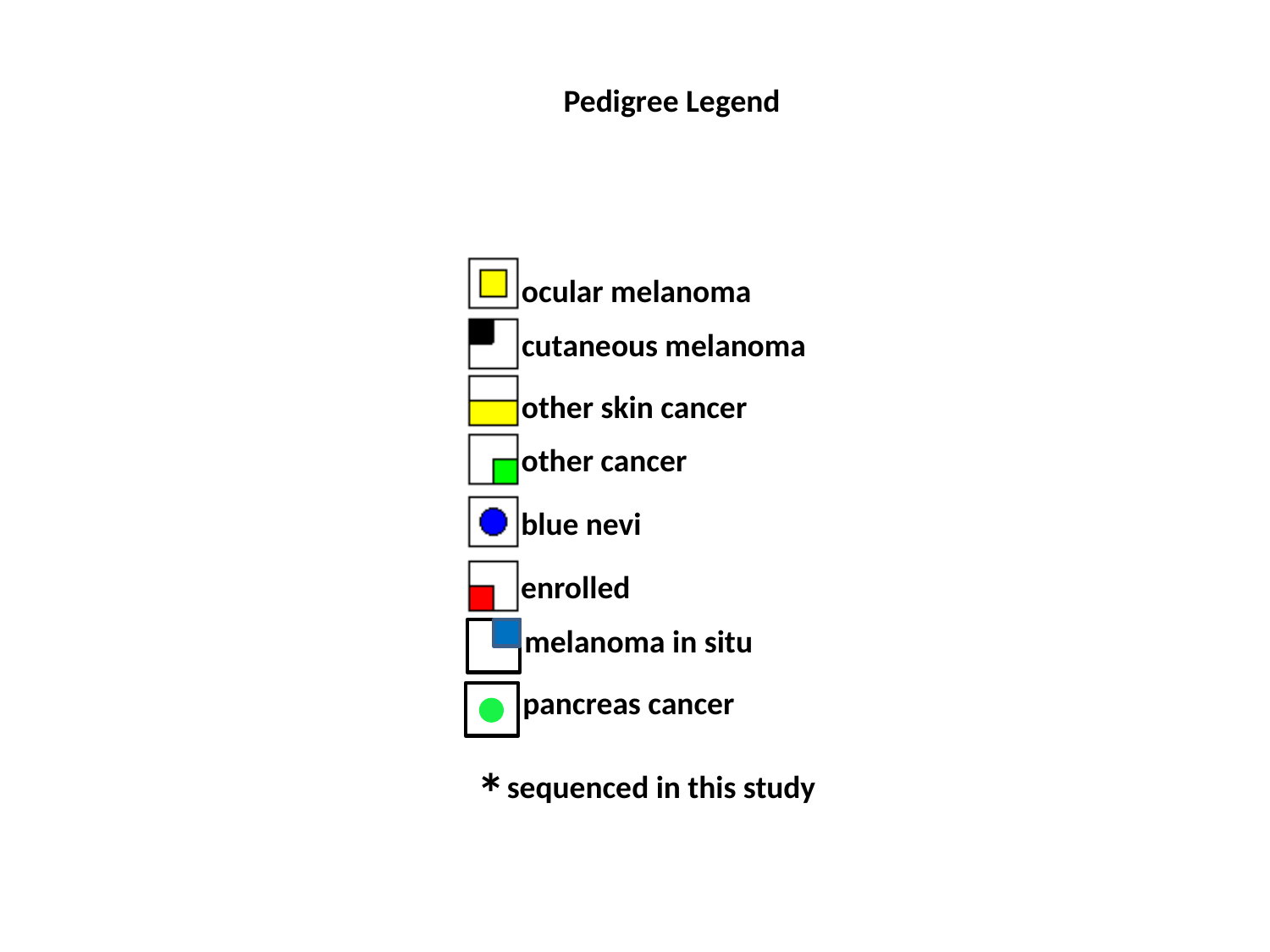

Pedigree Legend
ocular melanoma
cutaneous melanoma
other skin cancer
other cancer
blue nevi
enrolled
melanoma in situ
pancreas cancer
*
sequenced in this study

## Slide 2
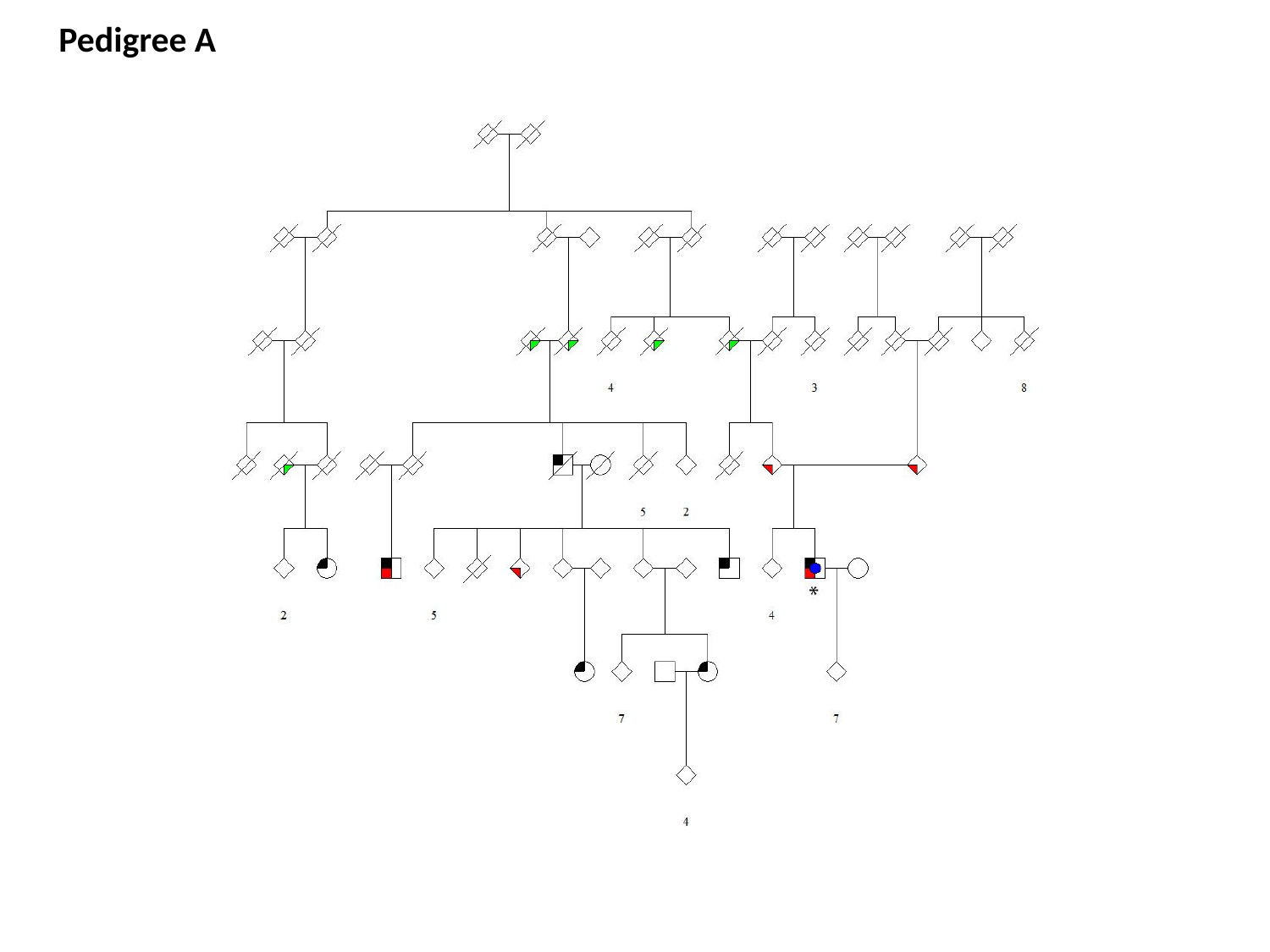

Pedigree A

## Slide 3
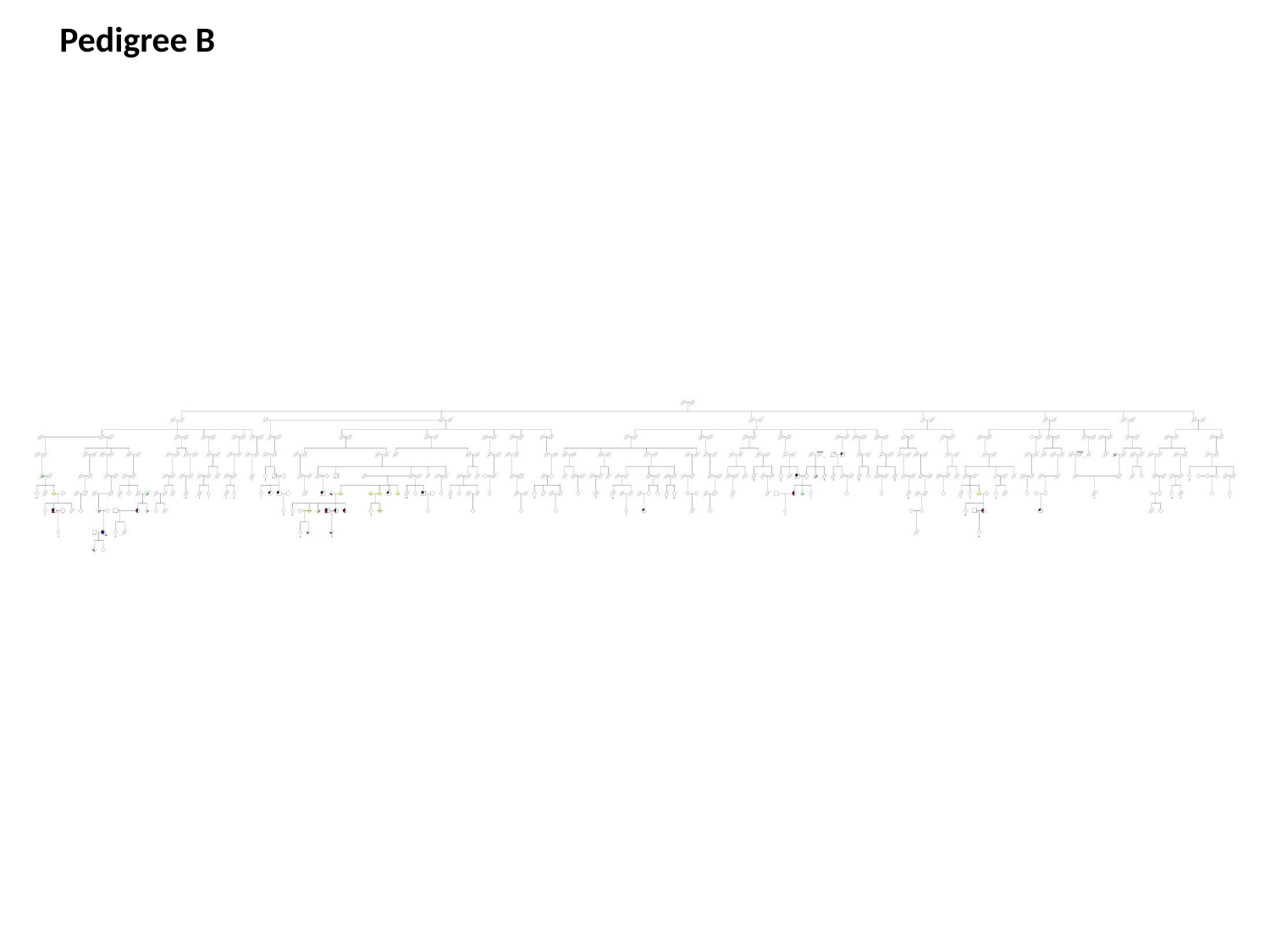

Pedigree B

## Slide 4
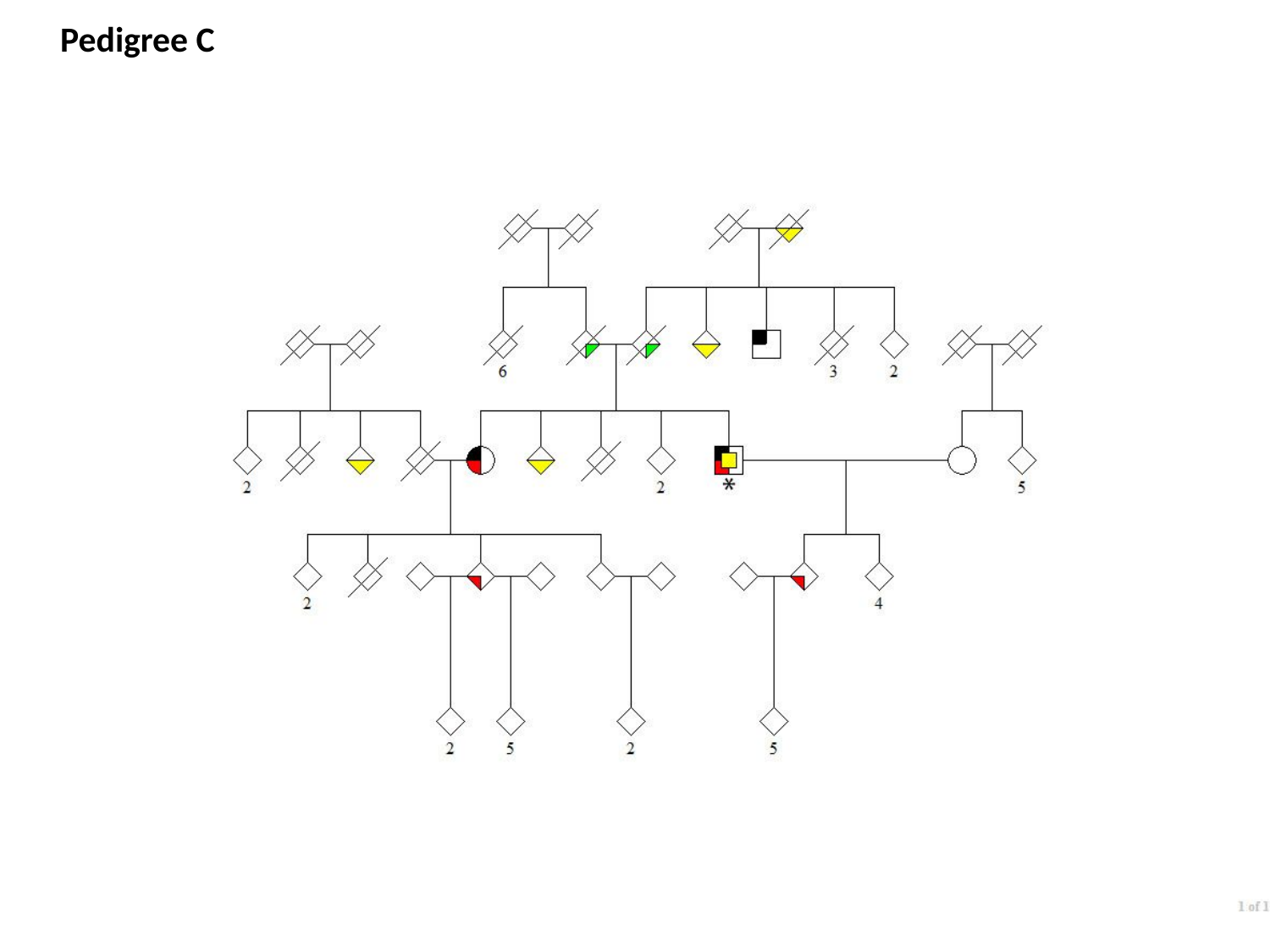

Pedigree C

## Slide 5
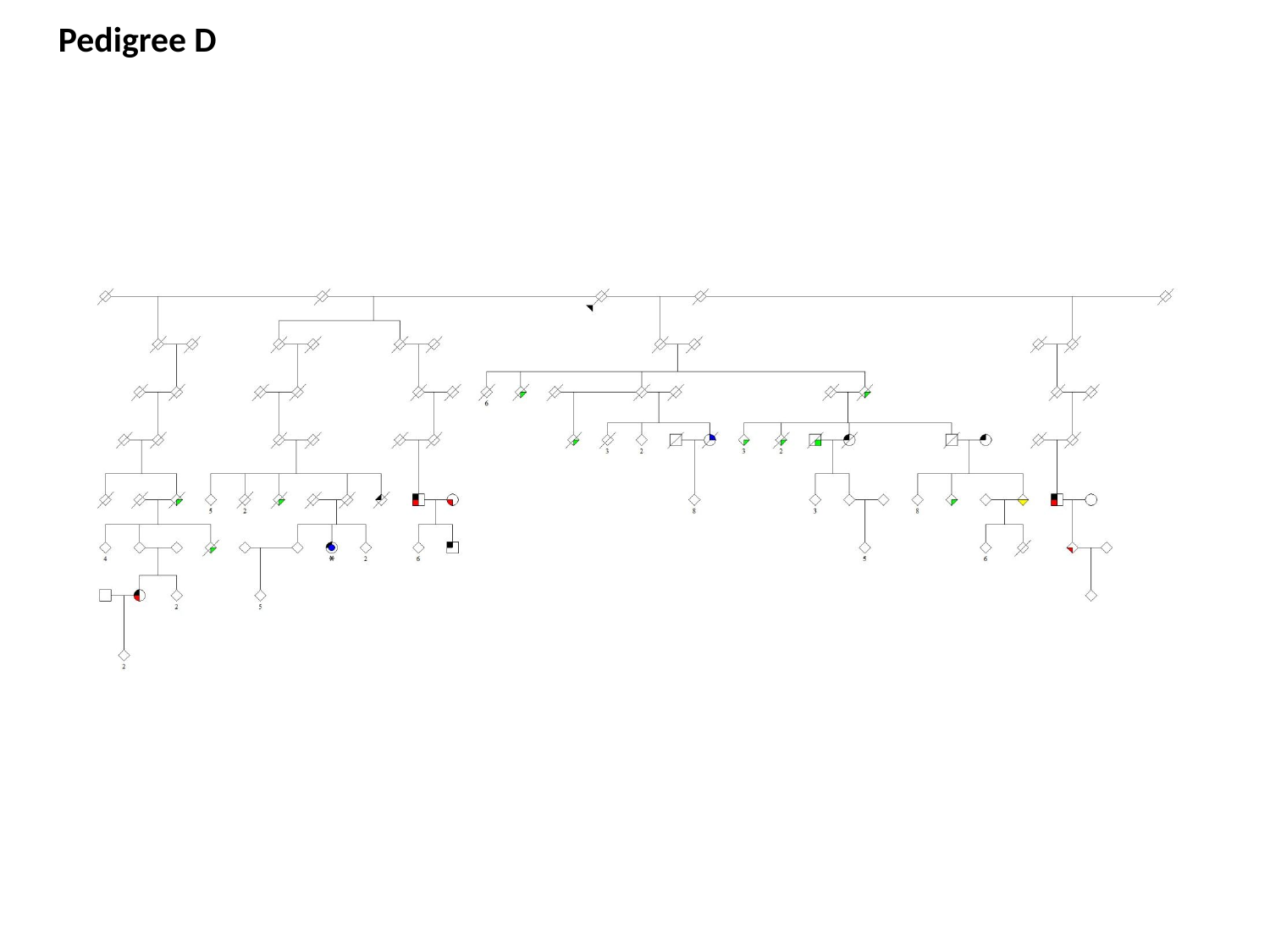

Pedigree D

## Slide 6
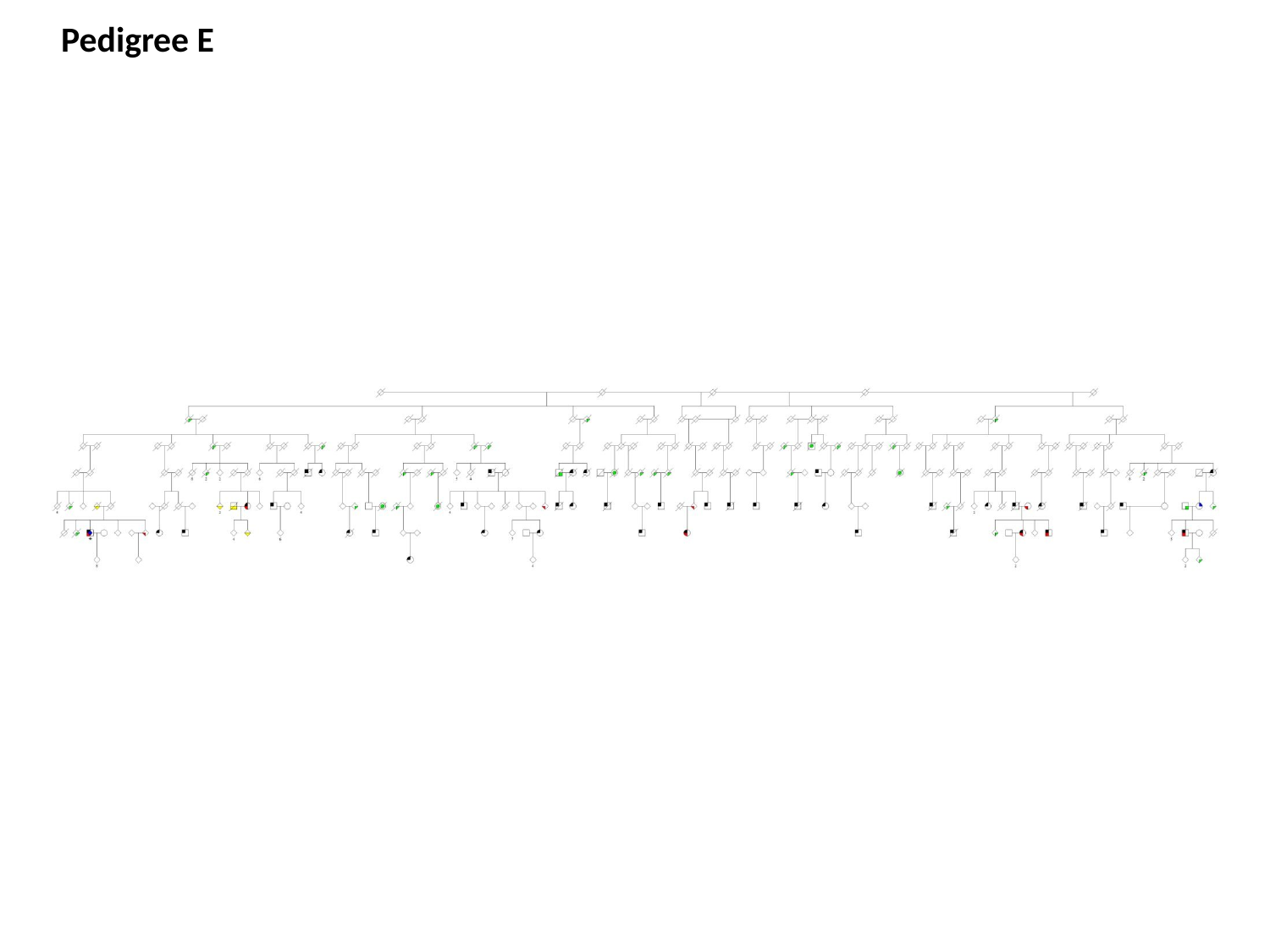

Pedigree E

## Slide 7
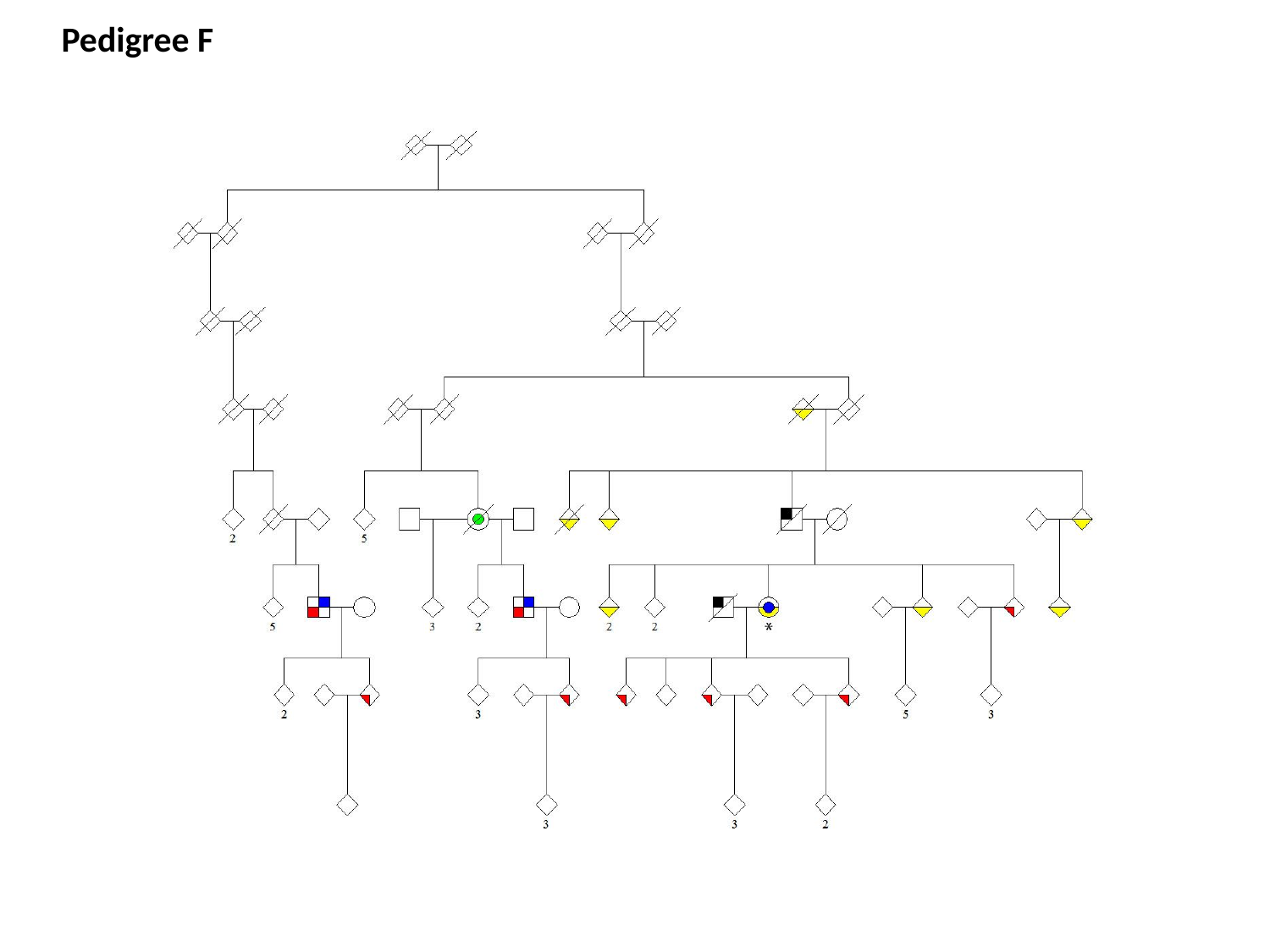

Pedigree F

## Slide 8
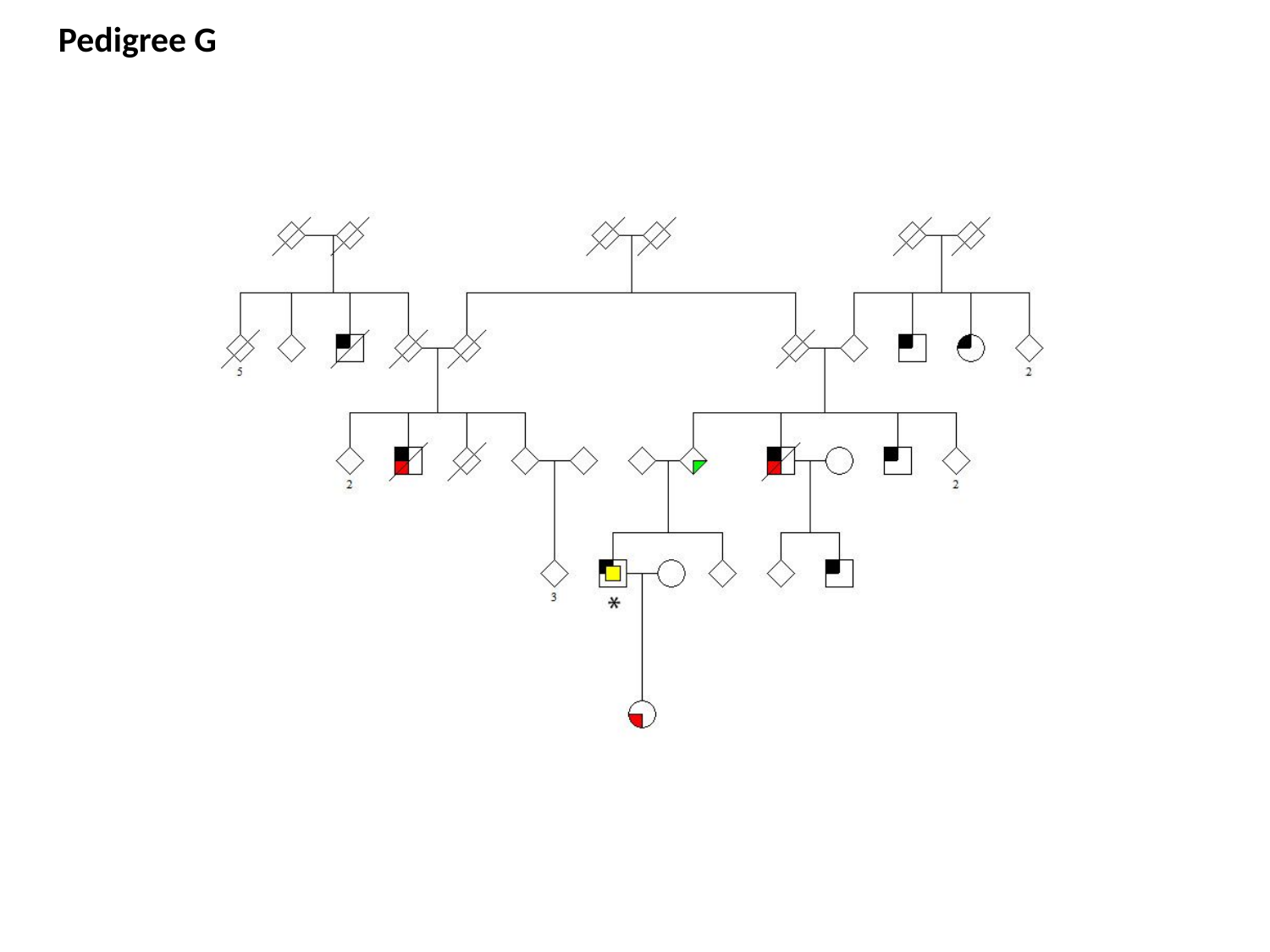

Pedigree G

## Slide 9
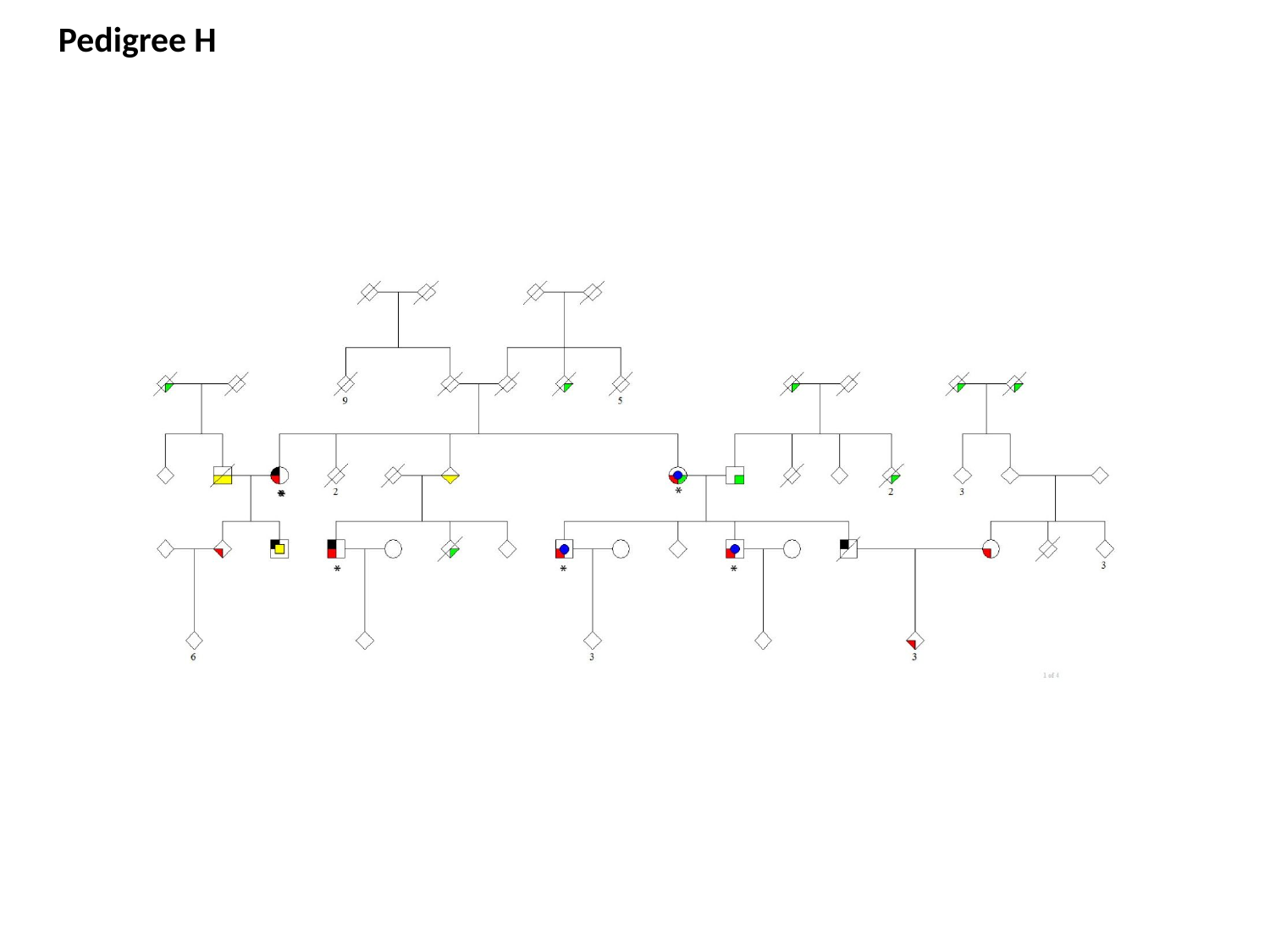

Pedigree H

## Slide 10
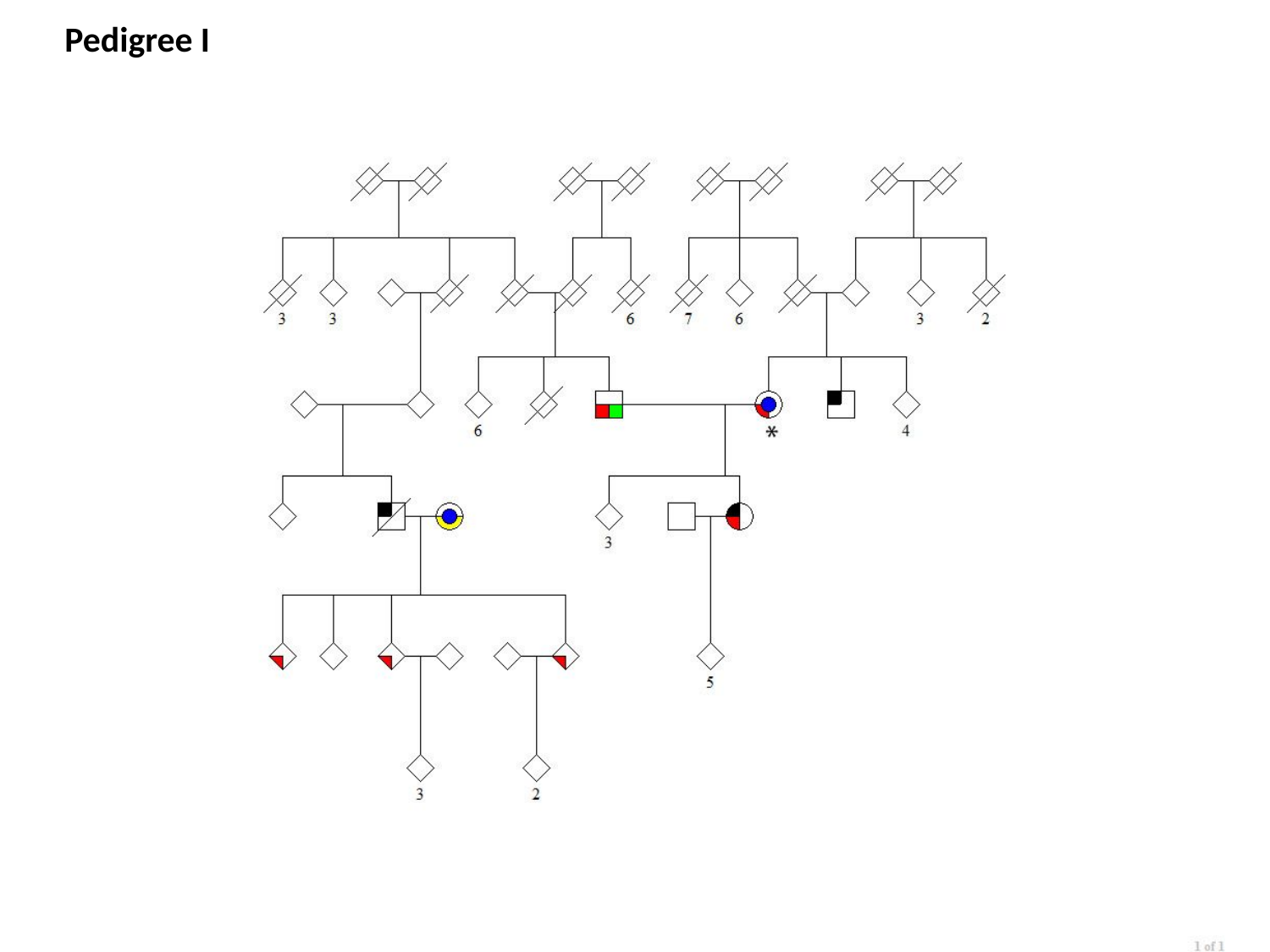

Pedigree I

## Slide 11
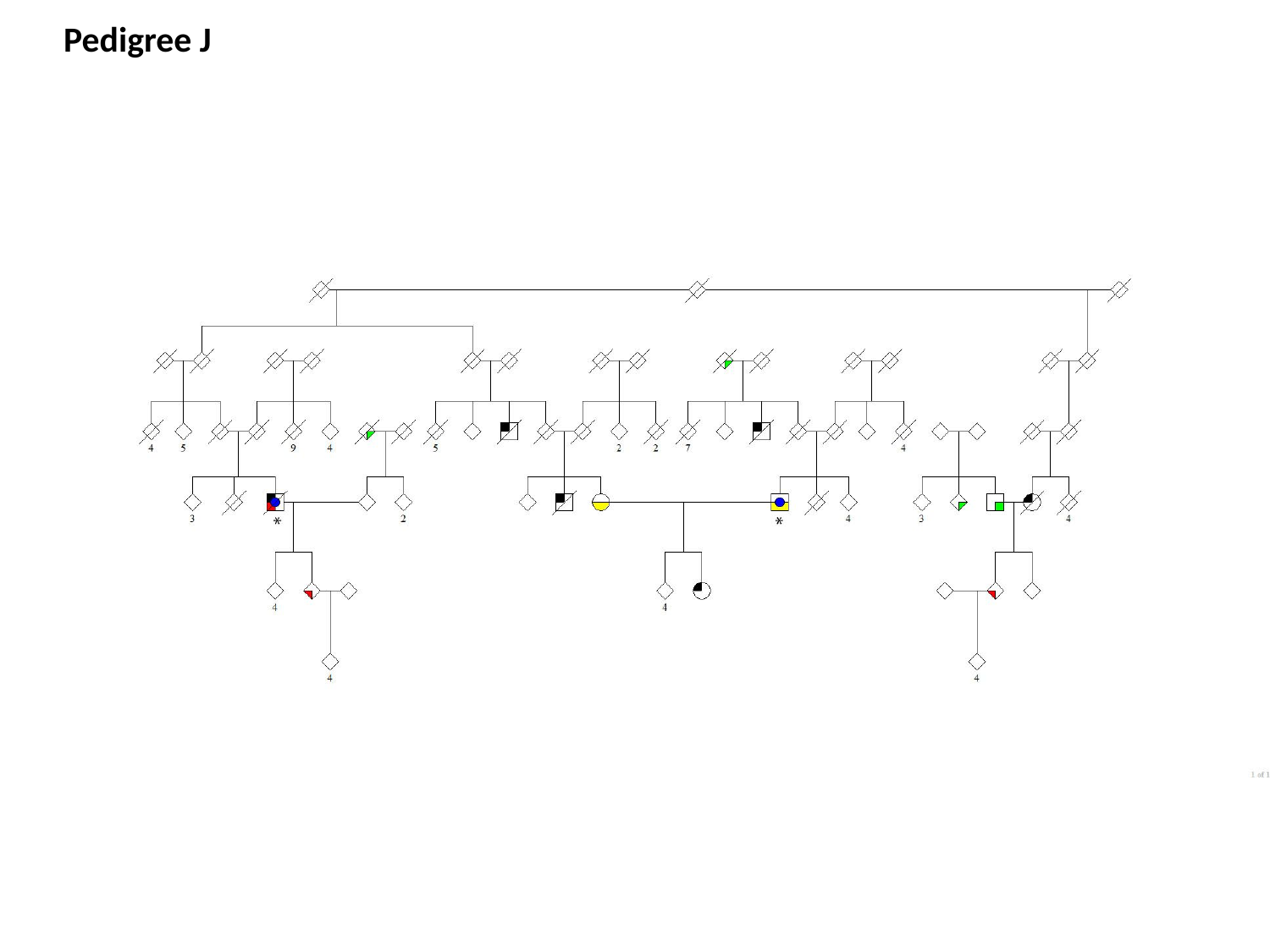

Pedigree J

## Slide 12
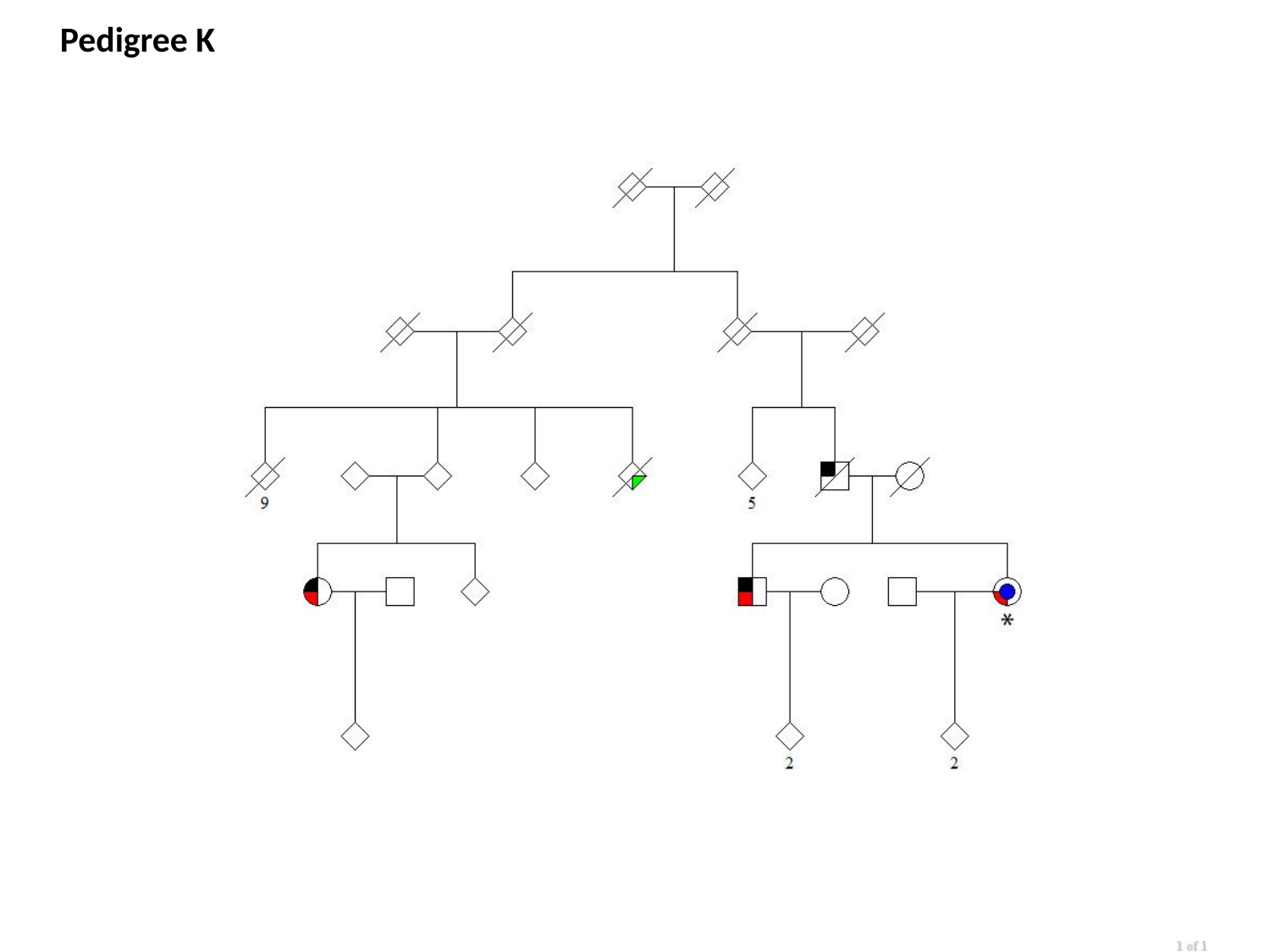

Pedigree K

## Slide 13
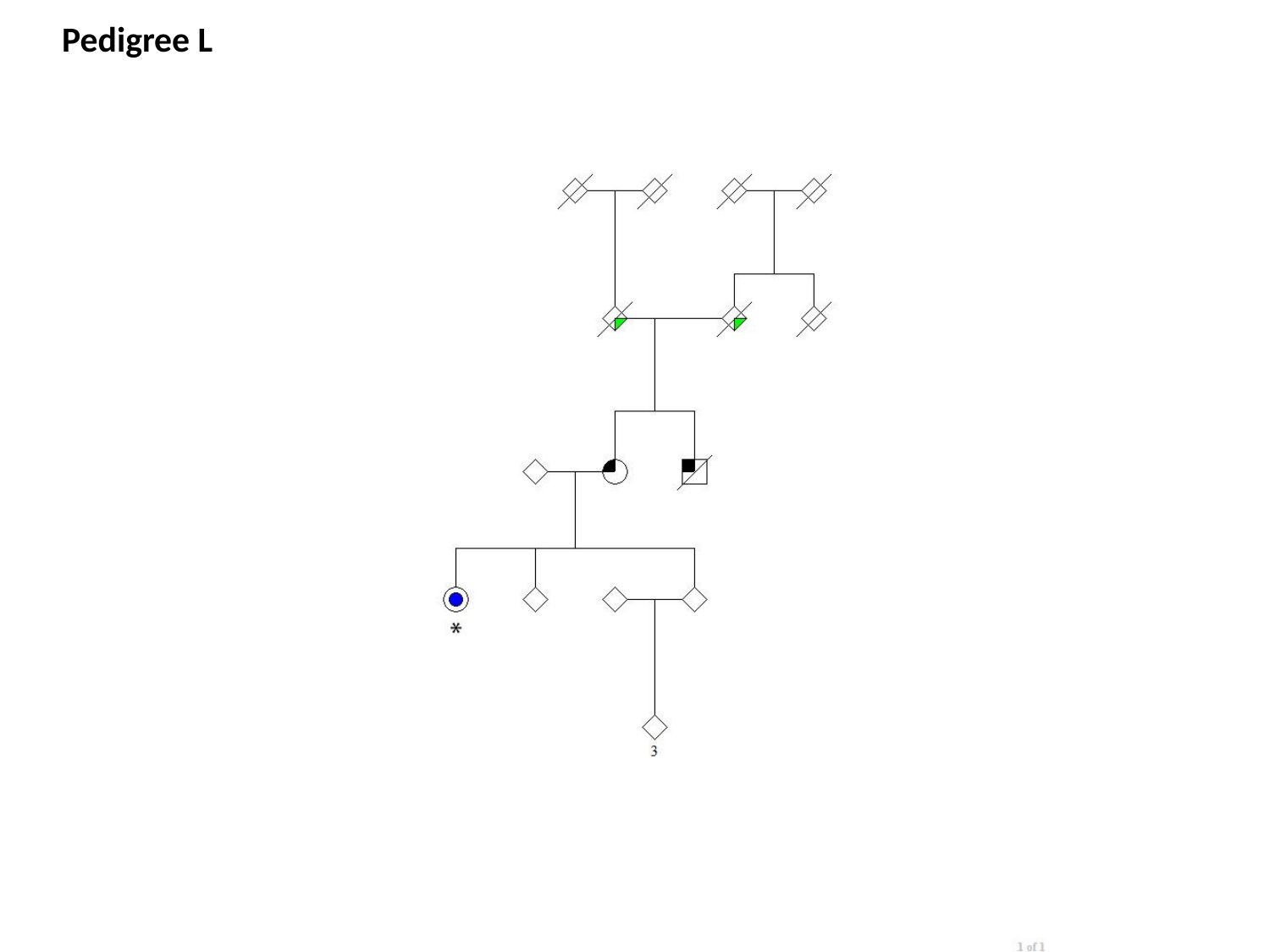

Pedigree L

## Slide 14
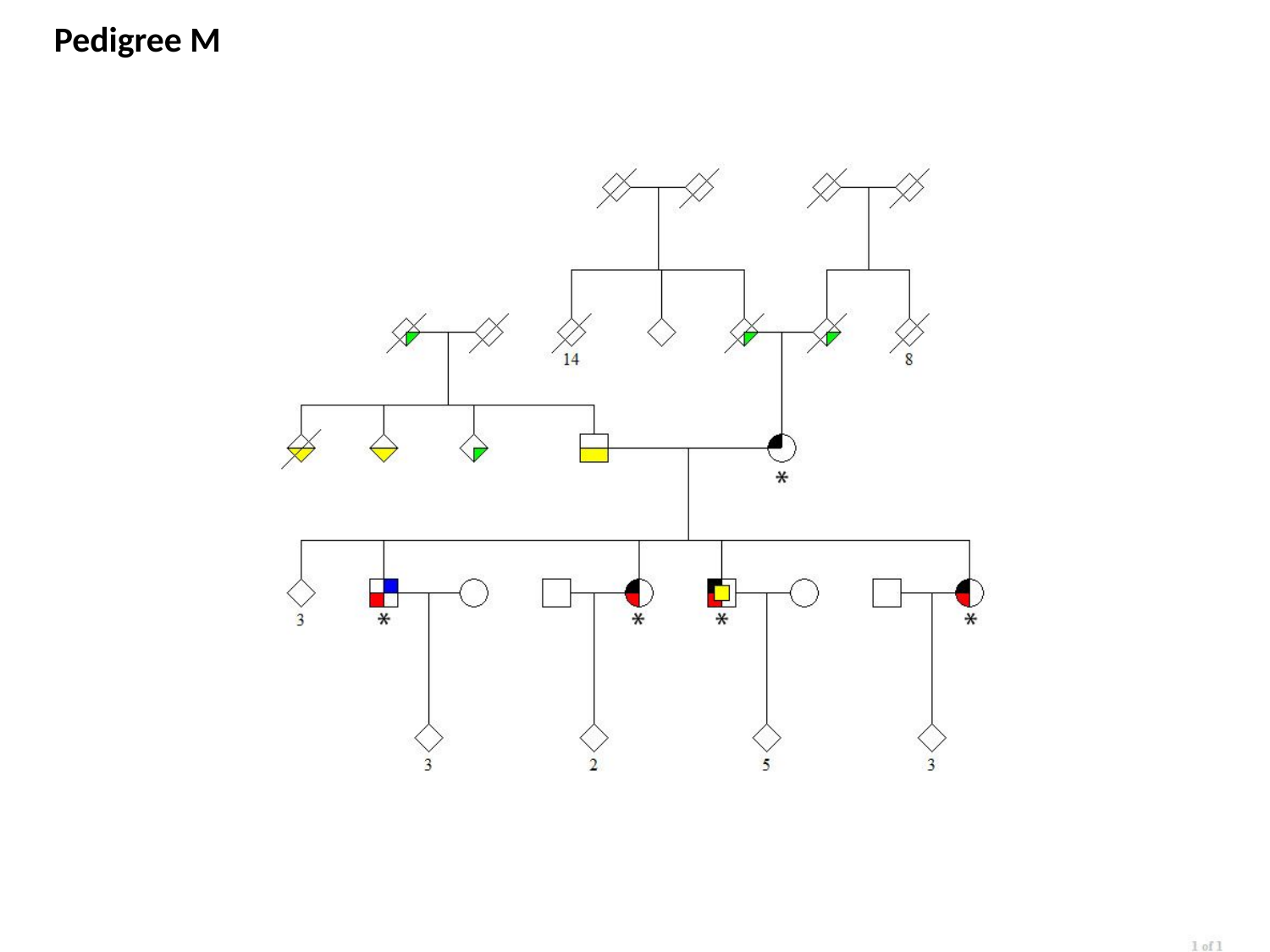

Pedigree M
